# Sex differences in reward network activation are linked to problematic substance use among high-risk adolescents

**DOI:** 10.3389/adar.2025.14591

**Published:** 2025-12-04

**Authors:** Olivia K. Murray, Paola P. Mattey-Mora, Joseph Aloi, Sydney Lovins, Michael P. Smoker, Leslie A. Hulvershorn

**Affiliations:** 1 Department of Psychiatry, Indiana University School of Medicine, Indianapolis, IN, United States; 2 Adolescent Behavioral Health Research Program, Indiana University School of Medicine, Indianapolis, IN, United States; 3 Medical Scientist Training Program, Indiana University School of Medicine, Indianapolis, IN, United States

**Keywords:** sex differences, adolescent substance use, risky decision-making, externalizing psychopathology, functional neuroimaging

## Abstract

**Background:**

Adolescents with externalizing (EXT) disorders, such as attention-deficit/hyperactivity disorder and conduct disorder—characterized by impulsivity and rule-breaking, are at elevated risk for substance use disorders (SUDs), partly due to deficits in risky decision-making. Sex differences in this association are understudied. Neuroimaging research shows females and males with EXT disorders exhibit different brain activation patterns during risky decisions. This study will explore how these sex differences relate to the development of problematic substance use in youth with EXT disorders.

**Method:**

A total of 115 (78 males, 37 females) drug-naive adolescents with EXT psychopathology performed the Balloon Analogue Risk Task (BART) during magnetic resonance imaging to assess risky decision-making brain activation. Then, participants and their guardians completed questionnaires at 6-month intervals to assess problematic substance use. Statistical analyses evaluated sex differences in brain activation—both parametrically modulated and unmodulated—within *a priori-*selected regions associated with risky decision-making and problematic substance use, using Cox proportional hazards models.

**Results:**

Higher modulated brain activation (as explosion probability increased) during the choice phase contrast, Choose Inflate—Choose Win, was associated with a lower hazard of problematic substance use in the right nucleus accumbens (Hazard Ratio (HR) = 0.68, 95% CI [0.49, 0.94], *p* = 0.01). This association was significant for females, but not for males, with the hazard ratios being significantly different between sexes. In the right nucleus accumbens, higher unmodulated choice phase activation in males was associated with lower hazard of problematic substance use (HR = 0.60, 95% CI [0.37, 0.97], *p* = 0.03); and in the right subgenual anterior cingulate cortex, higher unmodulated activation in this same contrast in females was associated with a lower hazard of problematic substance use [HR = 0.49, 95% CI (0.24, 0.97), *p* = 0.03].

**Conclusion:**

This study offers insight into sex differences in risky decision-making neural mechanisms and SUD risk among youth with EXT disorders. Our findings suggest typical risk signaling in the reward-processing network (nucleus accumbens and subgenual anterior cingulate cortex) may protect against substance use, particularly in females with EXT disorders. These findings emphasize the need for further sex-specific research and interventions for youth with EXT disorders.

## Highlights

- This study reveals sex differences in brain activation during a risky decision-making task, showing that greater activation in the right nucleus accumbens and subgenual anterior cingulate cortex is linked to a lower hazard of problematic substance use in females with externalizing disorders.
- These findings underscore the importance of understanding sex-specific neural mechanisms in substance use risk, suggesting that targeted interventions for females could focus on enhancing normal reward- and loss-processing systems.
- This study emphasizes the need for sex-specific approaches in substance use disorder prevention, as it demonstrates that neural mechanisms related to risky decision-making and reward processing may operate differently in males and females at high-risk for substance use.

## Introduction

Adolescents with externalizing (EXT) disorders—including attention-deficit/hyperactivity disorder (ADHD), oppositional defiant disorder, and conduct disorder—are an important group in which to investigate the development of substance use disorders (SUDs). Due to EXT disorders’ characteristic impulsivity, hyperactivity, aggression, and rule-breaking, risky/disinhibited decision-making is a common feature of these disorders which may increase risk for SUD [1–6]. Several longitudinal studies have shown increased SUD and substance problems for youth with EXT traits/symptoms, but these studies are limited to male samples or have not explored sex differences in how these psychopathologies relate to differences in SUD outcomes or risk [7–11]. This prevents us from truly characterizing risk profiles and interventions for females with EXT disorders.

Due to their propensity toward disadvantageous decisions and lower-probability, high-reward choices [12–15], adolescents with EXT disorders’ brain activity during risky decision-making is a promising aspect to evaluate in relation to their SUD risk profile. Indeed, neuroimaging studies have found sex differences in youth with EXT disorders’ risky decision-making brain activation. Females with EXT disorders have shown more activation before risky choices in the middle frontal pole than males with EXT disorders, a region of the frontoparietal network, involved with executive function [16, 17]. Given these sex differences and how risky decision-making deficits could contribute to increased risk for SUD, it is imperative to investigate how sex differences in youth with EXT disorders’ brain activation during risky decision-making may relate to sex differences in subsequent substance use.

To address that gap, this study examines brain activity during risky decision-making in youth with EXT disorders in regions where prior research has shown differential activation in individuals with ADHD a family history of SUD, as both are associated with heightened risk for SUD [18–21]. Additionally, activation in the regions examined has previously been linked to future substance use. Further, in the *a priori* selected regions, sex differences in youth with EXT disorders’ brain activation have yet to be explored in the context of SUD risk. The brain regions which satisfy these criteria include the nucleus accumbens (NAc) and the subgenual anterior cingulate cortex (sgACC).

The NAc has been extensively implicated in adolescent risk-taking and is a critical brain region for reward processing: spontaneous NAc activity predicts activity in other reward circuit nodes [22], and risky decision-making is associated with greater NAc activation in both the outcome/reward-receiving phase [23] and during the decision phase where heightened sensitivity to reward correlates with stronger NAc responses [24]. In both subjects with ADHD and those with family history of SUD, hypoconnectivity in the frontostriatal network, between the NAc and prefrontal cortex, has been found, representing the brain basis for dysregulated prefrontal control over subcortical reward-related regions [25–28]; and those with family history of SUD show blunted/reduced NAc activation during risky decision-making, particularly during reward anticipation [29, 30]. Moreover, further connecting these at-risk populations, many studies examining NAc activity in those with SUD family history have shown that NAc activation correlates with EXT behaviors [28–31]. NAc activation has been shown to predict future substance use with lower fronto-NAc connectivity relating to earlier drinking behavior [32] and greater NAc activation during reward anticipation predicting more and earlier substance use [33, 34]. Although these findings may initially appear to contradict the previously discussed comparisons between at-risk groups and controls, they align when considering that increased EXT behavior is linked to heightened NAc activation. One study reported sex differences where NAc volume was indirectly linked to future drinking through sensation seeking in males, but not females, with a positive association between volume and sensation seeking in males [35]. In our sample, we therefore hypothesize that for boys with EXT disorders compared to girls with EXT disorders, greater NAc activation when making risky choices (anticipating reward) will be associated with a higher hazard of problematic substance use.

The ACC has been shown to have functions related to loss avoidance—assessing how likely an error is and how problematic the consequences could be if the event occurs, guiding decisions to avoid risky situations, highlighting this area's function in conflict monitoring/cognitive control [36–39]. The ventral area of the ACC, encompassing the rostral ACC, sgACC, and pregenual ACC, is more specifically involved in loss avoidance in decision-making through its role in emotional and motivational processes of reward evaluation due to its links with the OFC, amygdala, NAc, and the limbic system [40, 41]. While the sgACC is best-known for its involvement in negative affect and depression [42–46], it is precisely this role in processing aversive stimuli and negative emotions that also links it to risk-reward processing: sgACC activity has been linked to uncertainty and under confidence in the decision-making process [45, 47–50]. Further, greater sgACC activation is frequently seen during exposure to substance cues and is associated with increased craving for substances [51, 52]. Relatedly, when participants are able to effectively control their cravings, there is greater activity in areas of the brain linked to cognitive control (dorsomedial, dorsolateral, and ventrolateral prefrontal cortex) paired with less activity in the sgACC—highlighting its craving-associated function [53]. A similar relationship was found in resting-state synchrony analyses which revealed that the connectivity between the inhibitory control network (dlPFC) and the sgACC and NAc increased as individuals progressed from short-term to long-term abstinence from alcohol [54, 55]. This suggests that in alcohol recovery, decision-making networks may helpfully become less synchronous with regions linked to appetitive drive and more synchronous with inhibitory control areas [54, 55]. Further, our group has found that youth at high risk for SUD related to diagnosed EXT disorders and family history of SUD showed greater sgACC area activation during reward loss (negative outcome) compared to healthy control youth [56]. Lastly, patients with alcohol use disorder compared to healthy controls showed greater activation in the sgACC during anticipation of monetary reward [57]. In conclusion, the sgACC appears to play a critical role in the interaction between risky decision-making and substance use. Therefore, in our sample, where we expect boys with EXT disorders will display greater risky and substance-use aligned behavior, we hypothesize that for boys, greater activation in the sgACC during reward anticipation and reward loss will be associated with greater problematic substance use.

Building on our examination of neural correlates underlying risky decision-making and their relevance to predicting problematic substance use, it is also critical to consider behavioral variables—specifically those modeling reward sensitivity and loss aversion. These constructs offer valuable insight into individual differences in decision-making processes that may predispose individuals to substance-related risk in a sex-specific fashion. Prior research has consistently demonstrated that individuals exhibiting heightened reward sensitivity [58–61] and reduced loss aversion [62–67] are more likely to engage in risk-taking behaviors and experience greater levels of substance use. Importantly, emerging evidence points to sex differences in these behavioral tendencies, with males typically displaying higher reward sensitivity [68–71] and lower loss aversion [67, 72–74] compared to females. We will investigate how these differences among youth with EXT disorders may help explain sex-specific patterns in substance use vulnerability. We hypothesize that higher reward sensitivity and lower loss aversion, particularly among males, will be associated with higher rates of problematic substance use.

Prior findings of sex differences in risky decision-making brain activity among youth with EXT disorders has yet to be evaluated in its relation to sex differences in substance use outcomes, despite this possibly helping explain sex differences in substance use patterns. We investigated these associations in youth with EXT disorders of ADHD and a disruptive behavior disorder via the brain activation in two regions of interest: the NAc and sgACC—crucial nodes in the reward-processing network [41, 75–77]. We hypothesized that greater NAc activation when making risky choices, and greater sgACC activation during reward anticipation and loss, would be associated with greater problematic substance use in boys compared to girls. The overarching hypothesis for this study is that for boys with EXT disorders compared to girls with EXT disorders, greater activation in reward network areas when making risky choices, anticipating rewards, and reacting to loss is associated with higher hazard of problematic substance use. In sum, within the context of EXT psychopathology as a risk phenotype for SUD, sex differences are under-investigated and how sex differences in risky decision-making relate to future risky substance use has yet to be explored. With this study, we are poised to explore this gap in a sample of 11–12-year-olds at-risk for SUD by virtue of their EXT psychopathology via neuropsychological underpinnings of risky decision-making.

## Methods

### Study design

The data for this study were collected as part of an ongoing longitudinal study in which youth were recruited from urban, suburban, and rural areas in Indiana in order to investigate the brain and behavioral basis of the development of risky behaviors [17, 56, 78–80]. This sample has been used in previously published studies (see: [17, 56, 78–83]), and several aspects—particularly those related to sex differences—have already been examined in earlier work from our lab [17, 81, 83]. For this manuscript, we are focusing on youth with EXT psychopathology to address a gap in the literature where most research has centered on healthy youth [32] or those with a family history of SUD [33, 34, 84]. In doing so, prior research has failed to explore how neural processing deficits in risky decision-making contribute to risk of developing problematic substance use in youth with the risk phenotype of EXT disorders, specifically. Each eligible participant completed an MRI session as well as behavioral and psychiatric assessments at a baseline assessment at age 11–12 years. The MRI session, which only occurred at the baseline visit, included anatomical and functional MRI (fMRI) scans to assess functional connectivity measures and brain responses during risky decision-making tasks. Following their baseline imaging, participants were followed up into later adolescence with behavioral questionnaires every 6 months assessing substance use outcomes as discussed below in the Measures section.

### Sample

**FIGURE 1:**
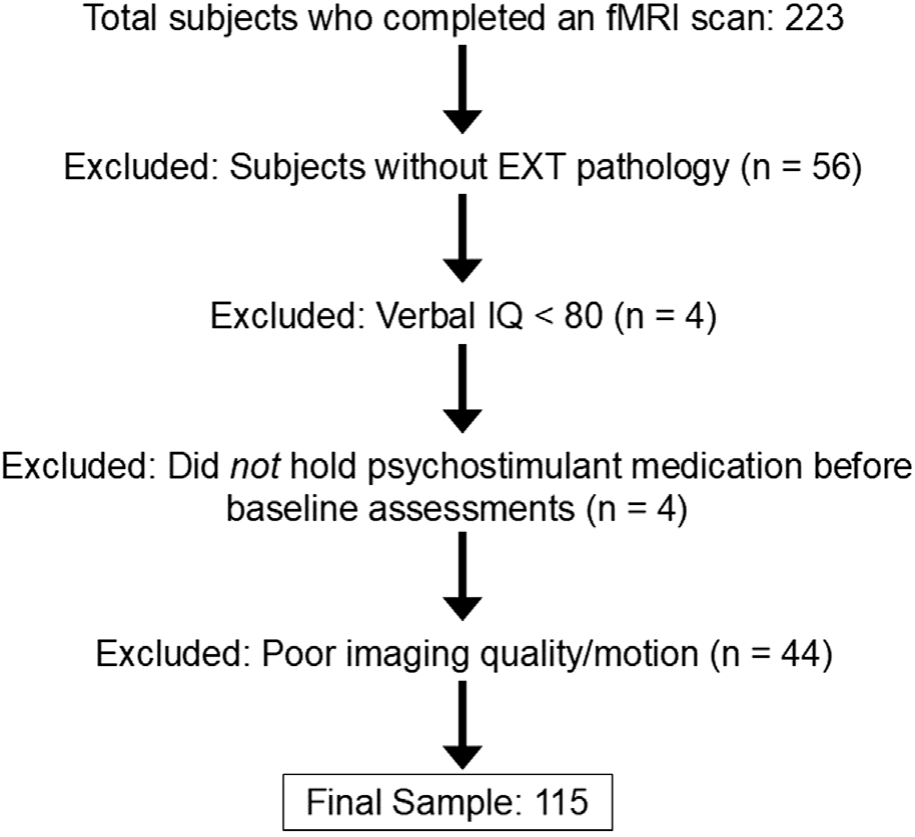
Flowchart of reasons for participant exclusion.

A total of 223 right-handed, English-speaking drug-naive 11–12-year-old participants and their guardians completed behavioral and psychiatric assessments. Each child’s biological sex was reported as male or female by the adult guardian at baseline. Exclusion criteria at baseline were: 1) lifetime history of any substance use or SUDs, psychotic symptoms, bipolar disorder, or autism spectrum disorders, 2) current DSM 5-defined major depressive disorder, 3) neurological disorder history (e.g., brain tumors, epilepsy, traumatic brain injury), 4) Full Scale or verbal IQ < 80 (to ensure comprehension of tasks and questionnaires), 5) active debilitating medical conditions, 6) maternal substance use during pregnancy, 7) MRI-related contradictions, 8) left-handedness, 9) siblings in the study. Figure 1 displays the consort diagram explaining reasons for subject exclusions and numbers eliminated for each reason. All procedures were conducted with approval from the Indiana University Institutional Review Board.

Of those who completed an imaging session, 167 were classified as high-risk for SUD due to EXT psychopathology and were considered for this study (see Figure 1). Youth with EXT disorders met criteria for DSM-5 defined diagnoses of ADHD and a disruptive behavior disorder: oppositional defiant disorder, conduct disorder, or unspecified disruptive behavior disorder. Participants taking psychostimulant medications were asked to refrain from taking their medication on study visit days, and a urine drug screen on each visit was conducted to verify substance-naive status. Numbers of participants being treated with psychostimulant medications at the time of baseline assessment are presented in Table 1. The final sample included in analyses consisted of 115 total subjects with EXT disorders—78 males and 37 females (Table 1; Figure 1).

**TABLE 1:** Demographics.

Variable	Females with EXT disorders (n = 37)	Males with EXT disorders (n = 78)	*p*-values
Age at visit 1/M (SD)	12.08 (0.53)	11.89 (0.56)	0.08
Gender diverse youth/N (%)	5 (13.51)	6 (7.69)	0.51
Family history of SUD (%)	21 (56.76)	36 (46.15)	0.38
Race/N (%)			0.48
White	20 (54.05)	49 (62.82)	
Black	13 (35.14)	19 (24.36)	
Multiracial	4 (10.81)	10 (12.82)	
Hispanic ethnicity/N (%)	3 (8.11)	7 (8.97)	1.00
Parental education max/N (%)			0.63
High school	4 (10.81)	8 (10.26)	
Some college/College	23 (62.16)	42 (53.85)	
Some graduate/Graduate	10 (27.03)	28 (35.90)	
SAVE TV/M (SD)	12.59 (1.07)	14.38 (5.42)	0.04
SAVE IV/M (SD)	24.76 (9.12)	30.64 (11.15)	<0.01
Parental monitoring/M (SD)	3.31 (0.69)	3.09 (0.73)	0.13
Stimulant med treatment at baseline/N (%)	13 (35.14)	38 (48.72)	0.24
PSU (any substance)/N (%)	12 (32.43)	18 (23.08)	0.40
PSU for alcohol/N (%)	4 (10.81)	3 (3.85)	0.29
PSU for E-cigarettes/N (%)	4 (10.81)	7 (8.97)	1.00
PSU for cannabis/N (%)	6 (16.22)	13 (16.67)	1.00
PSU for tobacco (smoke/chew)/N (%)	0 (0.00)	2 (2.56)	0.82

EXT, externalizing; SAVE, screen for violence exposure; TV, traumatic violence exposure subscale; IV, indirect violence exposure subscale; PSU, problematic substance use, defined by experiencing two or more consequences for one substance as reported by child or guardian.

#### Measures

##### Kiddie schedule for affective disorders and schizophrenia-present and lifetime version (KSADS-PL)

A semi-structured clinical interview, the Kiddie Schedule for Affective Disorders and Schizophrenia-Present and Lifetime Version (KSADS-PL) [85, 86], administered to guardians and youth, determined psychiatric diagnoses, including EXT diagnoses.

##### Substance use measures

Both youth and guardians completed virtual follow-up assessments at 6-month intervals after the baseline measurements, whenever feasible. Time points from our longitudinal follow-up included in this analysis ranged from 6 months to 84 months after the baseline MRI session. Substance use was assessed with the revised Drug Use Screening Inventory’s substance use domain (DUSI-R) [87] with additional categories covering more recent commonly used substances since the inventory’s release. The DUSI-R administered here includes 25 substance classes and assesses the frequency of monthly substance use (0 times, 1–2 times, 3–9, 10–20, 21+) and endorsement (yes/no) of DSM-5-based, substance-specific problems (e.g., Have you accidentally hurt yourself or someone else after using *substance in question*?). Our primary outcome of interest was adolescent problematic substance use, defined here as two or more self or guardian-reported DSM-5-based problems for any one substance. The outcome of interest, problematic substance use, was operationalized as the first time point at which the outcome was endorsed. Thus, the outcome was classified as either an event (child or guardian-reported problematic substance use) or as censored.

##### Family history of SUD

A covariate of significant interest associated with substance use risk is familial history of SUD due to its aforementioned genetic liability [20, 21]. Family History of SUD is defined here as the subject having a biological father *and* another first or second-degree family member with a past or present DSM-5-defined SUD (excluding isolated alcohol or tobacco use disorders). Maternal substance use during pregnancy was an exclusion criterion due to its potential confounding effects on adolescent development. As a result, collecting data on biological mothers with a history of SUD was not done. Instead, we included reports of SUDs in additional first- or second-degree relatives to capture a broader familial pattern of substance use risk, rather than focusing on maternal SUD.

##### Violence exposure

Violence exposure was included as a possible covariate due to the established positive association between adolescent violence exposure and future substance use [88, 89], as well as the likelihood of greater substance availability in high violence areas [90–94]. At baseline and in yearly follow-up surveys, adolescents completed portions of the Screen for Violence Exposure (SAVE) pertaining to subscales regarding their exposure to traumatic violence and indirect violence [95]. Questions belonging to the traumatic violence subscale include items like “Someone has pulled a gun on me” and “I have seen someone get killed” whereas indirect violence subscale items include “I have seen someone carry a gun” and “I have heard about someone getting killed.” Individual subscale scores were summed, with higher scores indicating greater violence exposure. Scores at baseline were used where available and then, if subjects were missing the baseline measure (n = 3), their next completed measure at a yearly survey was utilized (n = 1 at 1 year and n = 2 at 2-year survey).

##### Parental monitoring

At baseline, guardians described how frequently (i.e., Likert scale never to always) they monitor their children outside of school hours with 4 items adapted from the Silverberg Parental Monitoring Scale. Topics included knowing where their child is after school, calling/texting them to find out what they’re doing, scheduling their child’s time after school, and checking up on them when not in school. Their answers to these four items were averaged with higher final scores indicating more frequent parental monitoring. Parental monitoring was included as a potential covariate due to evidence that it causally affects adolescents’ substance use patterns [96].

##### Gender diverse youth

Gender is a social construct, where the term ‘cisgender’ refers to gender which aligns with sex assigned at birth. In this report we use the term, ‘gender diverse’ to describe youth whose self-reported gender identity (boy or girl), two or more times throughout the study period, did not align with their guardian-reported sex assigned at birth (reported at baseline; n = 11). While the relationship between gender identity and risky decision-making neural activation has not been directly studied, research has found that gender diverse individuals are more likely to experience higher rates of risky decision-making, impulsivity, and substance use compared to their cisgender peers—patterns often understood as responses to minority stress, social stigma, and systemic barriers [97–101].

### Procedures

#### Balloon analog risk task (BART)

**FIGURE 2:**
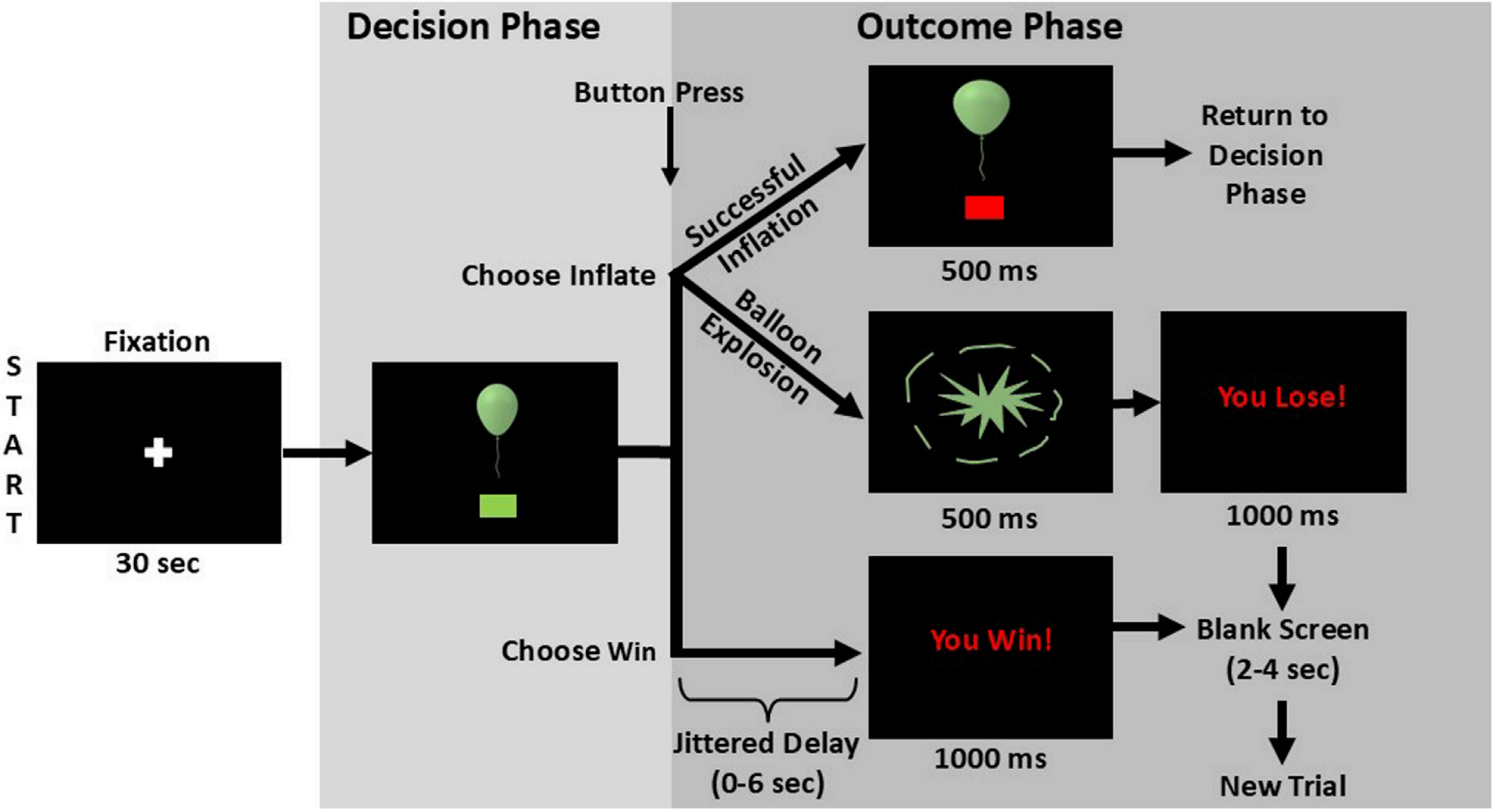
BART Schematic. After viewing a fixation cross, to begin the task sequence, a balloon appears alongside a green box prompt which indicates it is time to make a decision. Participants then press a button to either inflate the balloon once more (Choose Inflate) or to stop inflating the current balloon and bank the current worth of the balloon (Choose Win). If the participant chooses to inflate the balloon, the outcome will either be a successful inflation or a balloon explosion. In cases of successful inflation, the image of the now expanded balloon remains on screen for 500 milliseconds before the next decision prompt appears. If the balloon bursts, an explosion graphic is shown for 500 milliseconds followed by the message “You Lose!” for one second. If the participant selects “Choose Win,” a variable delay between zero and 6 seconds precedes the presentation of a “You Win!” message which is displayed for one second. The explode and win outcomes are followed by a fixation period of two to 4 seconds before a new trial is begun. Figure is adapted from Aloi et al. (2023) and reproduced from Murray et al. (2025), with permission.

During the imaging session, at baseline, participants completed MPRAGE and field mapping scans, followed three eight-minute fMRI-compatible BART scans [102, 103] to explore brain activation during risky decision-making. The BART models real-world risky choices and correlates with impulsivity, adolescent risk-taking, and SUDs [58–61, 104], making it a relevant tool for investigating sex influences on youth with EXT disorders’ risky decision-making. During the BART, participants decide between making a risky choice—risk losing accumulated cash rewards that increase each time a virtual balloon inflates, or a safe choice—banking the amount earned on a balloon before starting to inflate a new balloon (Figure 2). Participants are told to “inflate the balloon as much as you can without popping it” to earn money for each unexploded balloon and that they will win more money for larger balloons (paid in cash after scanning). Explosion probability increases parametrically with each inflation [102]. The parametric modulation of balloon explosion probabilities at each inflation are as follows: 0% for $0.0; 2.1% for $0.05; 4.2% for $0.15; 6.3% for $0.25; 14.6% for $0.55; 23.9% for $0.95; 31.3% for $1.45; 43.8% for $2.05; 56.3% for $2.75; 68.8% for $3.45; 79.2% for $4.25; 89.6% for $5.15. A jitter function is applied to the time between decision and outcome phase stimulus presentations of each trial to distinguish processes related to decision-making vs. feedback [102]. Participants were shown multiple virtual balloons and selected the appropriate button to either inflate the balloon (Choose Inflate), risking increasing cash for larger balloon size, or to stop inflating and save the money accumulated for that balloon (Choose Win). After choosing to inflate, the balloon either expands, increasing the reward for that balloon (Outcome Inflate), or it pops and the accumulated money for that balloon is lost (Outcome Explode). Excluding the initial inflation, explosions could occur at any balloon size, with increasing risk for larger balloons. When participants Choose Win, the accumulated rewards are banked, and a new balloon appears. During the three 8-min BART imaging sessions, participants could complete as many balloons as possible, with 12 inflations maximum per balloon. Before performing the BART in the MRI scanner, participants practiced on a desktop computer.

The BART behavioral variables recorded and analyzed included average adjusted pumps (the average number of inflations on unexploded balloons) and total inflations following explosions. The average adjusted pumps measure is considered the most reliable indicator of an individual’s reward sensitivity from the BART behavior measures with greater adjusted pumps representing greater sensitivity to reward [58–61, 105], while we propose total inflations after explosions can be interpreted as a measure of loss aversion, with fewer inflations after an explosion reflecting greater aversion to loss [102]. Behavior variables were averaged over a participant’s three BART sessions.

#### MRI data acquisition and preprocessing

Participants completed brain imaging in sessions which lasted less than 90 min on a research-dedicated 3.0-T Siemens Prisma MRI scanner with a 32-channel head coil. A high-resolution 3D magnetization prepared rapid gradient echo (MPRAGE) scan consisting of 160 sagittal slices and 1.05 × 1.05 × 1.2 mm^3^ voxels was completed. For BART runs, a T2*-weighted gradient echo-planar imaging (EPI) sequence was used (54 axial slices; voxel size 2.5 × 2.5 × 2.5 mm^3^; TR/TE 1200/29 ms; flip angle 65°; field-of-view 220 × 220 mm^2^; matrix 88 × 88), using a multiband sequence with a multiband factor of 3.

EPI scans were first distortion-corrected using fMRIB Software Library (FSL)’s “topup” tool, using distortion field estimates from the two opposite-phase spin echo field mapping. Anatomical scans were registered to Montreal Neurological Institute-152 (MNI152) standard MRI template brain volume space, and EPI data were aligned with each participant’s anatomical scan using afni_proc.py [106, 107], a comprehensive processing pipeline in AFNI (Analysis of Functional NeuroImages). Also, using afni_proc.py, functional images were motion-corrected and spatially smoothed with a 6 mm Gaussian kernel, and time-series normalization to a T1-weighted image was conducted, followed by an adjustment to a 100-scale per voxel. Lastly, unsupervised independent components analysis (ICA)-based denoising with ICA-AROMA [108, 109] from FSL’s MELODIC tool was used for robust data cleaning by identifying and removing noise-related components.

Visual inspection of brain activity ensured the presence of expected activation patterns corresponding to the task. This step involved examining the spatial distribution of activation maps to confirm typical task-related regions were engaged (e.g., visual cortex). Additionally, care was taken to avoid the occurrence of widespread negative activation, which could indicate global signal regression artifacts, head motion, or other preprocessing issues. Based on this evaluation, participants (n = 44) with unsatisfactory functional imaging were excluded from the analyses, with specific reasons for exclusions presented in the Supplementary Material.

### Neuroimaging data analysis of the BART

After preprocessing and noise reduction, runs were concatenated, and a general linear regression model (GLM) with random effects was used to estimate event-related responses in AFNI. *Choice events*, aligned to the repetition time (TR) that included the button press response, were modeled as Choose Inflate (choosing to continue inflating the balloon) or Choose Win (choosing to discontinue inflating and bank money) regressors. *Outcome events* were modeled as the TR that included balloon explosion (Outcome Explode), successful balloon inflation (Outcome Inflate), or the outcome of discontinuing inflations (Outcome Win). Balloon explosion probabilities were included as parametric modulators for each event-type regressor [e.g., Choose Inflate *P (explode), Outcome Inflate *P (explode)], except for Outcome Win, which has no uncertainty). Parametric modulators were incorporated to examine neural activation patterns that specifically track the escalating risk of both reward and loss as balloon size increases, providing insight into how brain regions dynamically respond to varying levels of decision-making uncertainty. To compare activation differences between conditions, individual subject activation maps were subjected to a voxel-wise subtraction using the 3dcalc tool in AFNI to compute the difference in activation between conditions, adjusting for individual differences in baseline activation. There were four contrast subtraction maps: Choose Inflate—Choose Win (modulated and unmodulated) and Outcome Explode—Outcome Inflate (modulated and unmodulated).

Choice phase contrast brain activation can be interpreted as brain activation during the approximately two second interval when participants made risky choices (Choose Inflate) compared to safe ones (Choose Win) via a button press, as those choices became riskier (parametrically modulated) or were averaged, ignoring risk of balloon explosion (unmodulated). Outcome phase/contrast brain activation is similar and refers to the two-second period when participants saw the result of their decision to inflate—either a balloon explosion or a successful inflation.

#### ROI mask creation and analysis

Separate regions of interest (ROIs) of the left and right NAc and left and right sgACC were selected as discussed in the Introduction due to their relevance for risky decision-making and SUD risk, and their masks were generated in AFNI using the MNI Glasser HCP atlas [110] for sgACC and the Brainnetome atlas [111] for NAc (Table 2). For each participant, we extracted the average activation within each region bilaterally for each of the four contrasts. To account for the broad range of activation across the four ROIs and facilitate comparison of activation between these ROIs, average brain activation was standardized by z-scoring for each ROI and contrast.

**TABLE 2:** *A Priori*-defined regions of interest (ROIs): Brodmann areas (BA), cluster sizes, and Montreal Neurological Institute (MNI) center of mass coordinates.

ROI	BA (if cortical)	Cluster size (voxels)	Center of Mass MNI coordinates (mm)		
			X	Y	Z
Left NAc	-	152	−17	4	−9
Right NAc	-	159	13	11	−9
Left sgACC	25/32	113	−5	21	−12
Right sgACC	25/32	118	3	20	−12

NAc, nucleus accumbens; sgACC, subgenual anterior cingulate cortex; cluster size reflects the number of voxels in each ROI.

### Statistical analyses

To investigate sex differences in the association between brain activation and the hazard of problematic substance use in youth with EXT disorders, we selected the two ROIs discussed above which are highly relevant in risky decision-making and SUD risk. Then we applied a Cox proportional hazards model for each ROI and for each contrast (e.g., parametrically modulated Choose Inflate—Choose Win). Standardized brain activation (z-scored), sex, and their interaction were modeled with time to problematic substance use event or censorship and presence of problematic substance use event in these models. Cox proportional hazards models were conducted using the survival package in R and hazard ratios (HR) with 95% confidence intervals (CI) are reported. With four contrasts and four ROIs, a total of sixteen models were conducted. To control the family-wise error rate, p-values were adjusted for multiple comparisons using the Hochberg procedure implemented in R. Significance threshold was set at *p* < 0.05.

Covariate selection was determined by a stepwise model selection procedure using the Akaike Information Criterion (AIC). The baseline model included average brain activation as the sole predictor of time to problematic substance use. A full model incorporated sex and the covariates described above which are related to adolescent development and substance use risk, including family history of substance use, traumatic violence subscale score from the SAVE questionnaire, and the average parental monitoring score. Using stepAIC from R’s MASS package [112], models for each contrast’s activation in each ROI were iteratively evaluated in both forward and backward directions to determine the most parsimonious model with the lowest AIC. Further details and results from stepAIC model selection can be found in the Supplementary Material.

As a secondary analysis, we explored how variables representative of loss aversion (total inflations after explosions) and reward sensitivity (average adjusted pumps) relate to the hazard of developing problematic substance use in the overall sample and then in subsamples of just females and males. We created two Cox proportional hazards models for problematic substance use, this time modeling the behavior variables as predictors. Then, we again conducted a Cox proportional hazards model for each ROI and contrast (e.g., parametrically modulated and unmodulated Choose Inflate—Choose Win) in separate samples of females and males, this time modeling the behavioral variables as interaction terms to assess potential effect mediation in the hazard models between neuroimaging contrasts and problematic substance use. Standardized brain activation (z-scored), the behavioral variable, and their interaction were included in the models, along with the covariates discussed above. Cox proportional hazards models were again conducted using the *survival* package in R, and hazard ratios (HR) with 95% confidence intervals (CIs) are reported for brain activation and the interaction between brain activation and the behavior variables for females and males in Supplementary Tables S4, S5. As in the main analysis, to control the family-wise error rate, p-values for each separate analysis, reward sensitivity and loss aversion, were adjusted for multiple comparisons using the Hochberg procedure implemented in R. Significance thresholds were set at *p* < 0.05.

To explore the effects of stimulant medication use on neural activation during decision-making and problematic substance use outcomes, we performed a sensitivity analysis. Participants were divided into two groups based on stimulant medication use by the time of the baseline visit: those currently taking stimulant medication (n = 51) and those who were not (n = 64). Mean standardized activation values were extracted for each participant across the four ROIs and four task contrasts, yielding a total of 16 comparisons. Independent samples *t*-tests were conducted to compare the stimulant and non-stimulant groups across these 16 ROI × contrast combinations. A chi-square test was also performed to assess group differences in the number of participants reporting problematic substance use.

Given the small number of gender diverse youth in our sample and to explore the possible effects of gender diversity on the association between sex, risky decision-making brain activation, and problematic substance use, we conducted a supplementary analysis. This analysis used the same parameters described above, adding gender (cisgender = 0 and gender diverse = 1) to the previously described models.

## Results

All hazard ratios and their corresponding confidence intervals for each ROI and contrast, uncorrected for multiple comparisons, are presented in Table 3. No findings remained significant after correction for multiple comparisons, therefore only preliminary, uncorrected results are presented and discussed below.

**TABLE 3:** Hazard ratios (HR) for activation (standardized) and problematic substance use.

ROI	Main HR (95% CI)	p*	Female HR (95% CI)	p*	Male HR (95% CI)	p*	Sex diff. HR *p*
**Choose inflate—choose win modulated**							
Left NAc	0.92 (0.61–1.41)	0.71	0.77 (0.44–1.34)	0.35	1.14 (0.64–2.03)	0.65	0.58
Right NAc	0.68 (0.49–0.94)	0.01	0.64 (0.44–0.92)	0.01	0.78 (0.43–1.41)	0.40	0.04
Left sgACC	0.94 (0.63–1.40)	0.75	1.18 (0.63–2.23)	0.59	0.81 (0.49–1.34)	0.40	0.61
Right sgACC	0.92 (0.65–1.30)	0.64	0.92 (0.58–1.46)	0.71	0.93 (0.56–1.54)	0.76	0.89
Choose inflate—choose win unmodulated							
Left NAc	1.02 (0.69–1.50)	0.92	1.05 (0.47–2.35)	0.91	1.01 (0.64–1.60)	0.96	0.99
Right NAc	0.67 (0.45–1.01)	0.05	0.84 (0.42–1.68)	0.63	0.60 (0.37–0.97)	0.03	0.10
Left sgACC	1.16 (0.80–1.70)	0.43	0.90 (0.51–1.59)	0.72	1.37 (0.86–2.18)	0.18	0.37
Right sgACC	1.12 (0.76–1.65)	0.56	0.49 (0.24–0.97)	0.03	1.47 (0.95–2.27)	0.08	0.02
Outcome explode—outcome inflate modulated							
Left NAc	1.29 (0.77–2.18)	0.33	2.00 (0.84–4.76)	0.11	0.98 (0.57–1.68)	0.94	0.29
Right NAc	1.26 (0.85–1.88)	0.25	1.55 (0.92–2.62)	0.10	0.88 (0.45–1.70)	0.69	0.24
Left sgACC	0.87 (0.60–1.26)	0.46	0.96 (0.56–1.65)	0.88	0.80 (0.48–1.34)	0.39	0.68
Right sgACC	0.88 (0.63–1.22)	0.44	1.11 (0.40–3.06)	0.84	0.86 (0.62–1.18)	0.34	0.62
Outcome explode—outcome inflate unmodulated							
Left NAc	1.02 (0.67–1.54)	0.92	1.02 (0.51–2.06)	0.94	1.02 (0.61–1.70)	0.94	0.99
Right NAc	1.15 (0.82–1.62)	0.41	1.24 (0.74–2.05)	0.41	1.09 (0.68–1.72)	0.72	0.67
Left sgACC	1.04 (0.76–1.42)	0.80	1.00 (0.70–1.43)	0.99	1.20 (0.62–2.32)	0.58	0.86
Right sgACC	0.99 (0.75–1.32)	0.96	1.02 (0.75–1.38)	0.90	0.89 (0.46–1.71)	0.73	0.93

Models were adjusted for family history of substance use, parental monitoring, and traumatic violence exposure; NAc, nucleus accumbens; sgACC, subgenual anterior cingulate cortex; problematic substance use is defined by experiencing two or more consequences for at least one substance as reported by child or guardian; p* = p-value uncorrected for multiple comparisons; sex diff HR *p*, the *p*-value indicating whether the hazard ratio (HR) differs significantly between males and females.

### Choice phase results

#### Parametrically modulated BOLD signal

**FIGURE 3:**
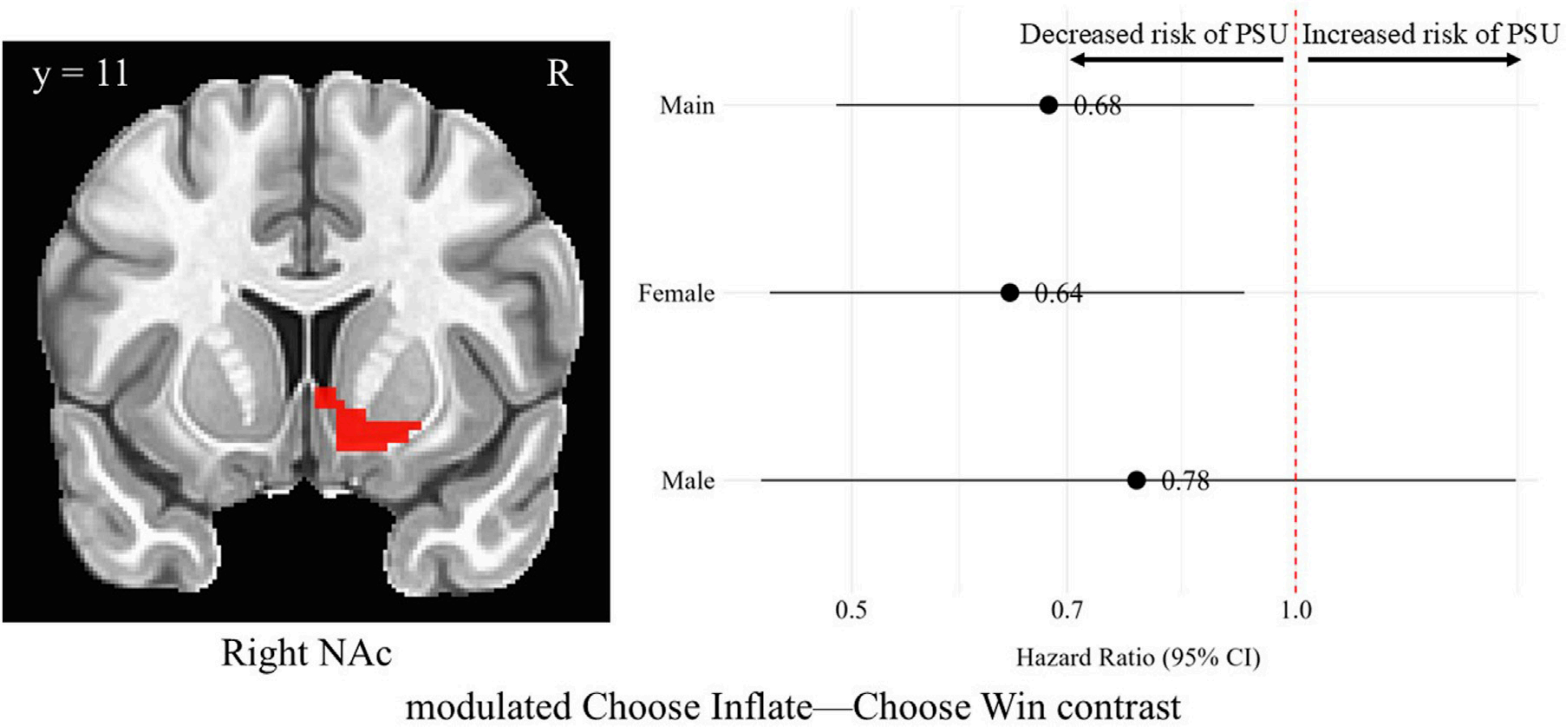
Hazard ratios for problematic substance use (PSU) risk based on modulated choice phase activation in the right nucleus accumbens. A coronal image of the right NAc cluster is depicted in the left side of the figure. On the right, each point represents the estimated HR for PSU risk per unit increase in activation, while the horizontal lines indicate the corresponding 95% CIs. A red vertical reference line at HR = 1.0 indicates no effect. Points to the right of this line suggest greater risk, whereas points to the left indicate lower risk of PSU. Statistically significant associations are indicated by confidence intervals that do not cross the reference line. Models were adjusted for family history of substance use, parental monitoring, and traumatic violence exposure.

Modulated Choose Inflate–Choose Win brain activation was significantly associated with problematic substance use in the right NAc. For every unit increase in standardized average brain activation the hazard of having problematic substance use decreased across all participants [HR = 0.68, 95% CI (0.49, 0.94), *p* = 0.01]. In this same region on the same modulated choice contrast, average brain activation (i.e., activation which increased with increasing probability of balloon explosion) in females was also significantly associated with a lower hazard of problematic substance use [HR = 0.64, 95% CI (0.44, 0.92), *p* = 0.01]. Males’ average activation was not significantly associated with problematic substance use [HR = 0.78, 95% CI (0.43, 1.41), *p* = 0.40]. Differences in hazard ratios were found between males and females (*p* = 0.04). However, none of these differences survived correction for multiple comparisons. There were no significant findings with parametrically modulated BOLD signal in the left NAc, left sgACC, or right sgACC. Figure 3 shows the right NAc region and a forest plot of hazard ratios with 95% confidence intervals for the overall sample, females, and males corresponding to the relationship between average activation intensities during the modulated Choose Inflate—Choose Win contrast and problematic substance use.

#### BOLD signal, without parametric modulation

**FIGURE 4:**
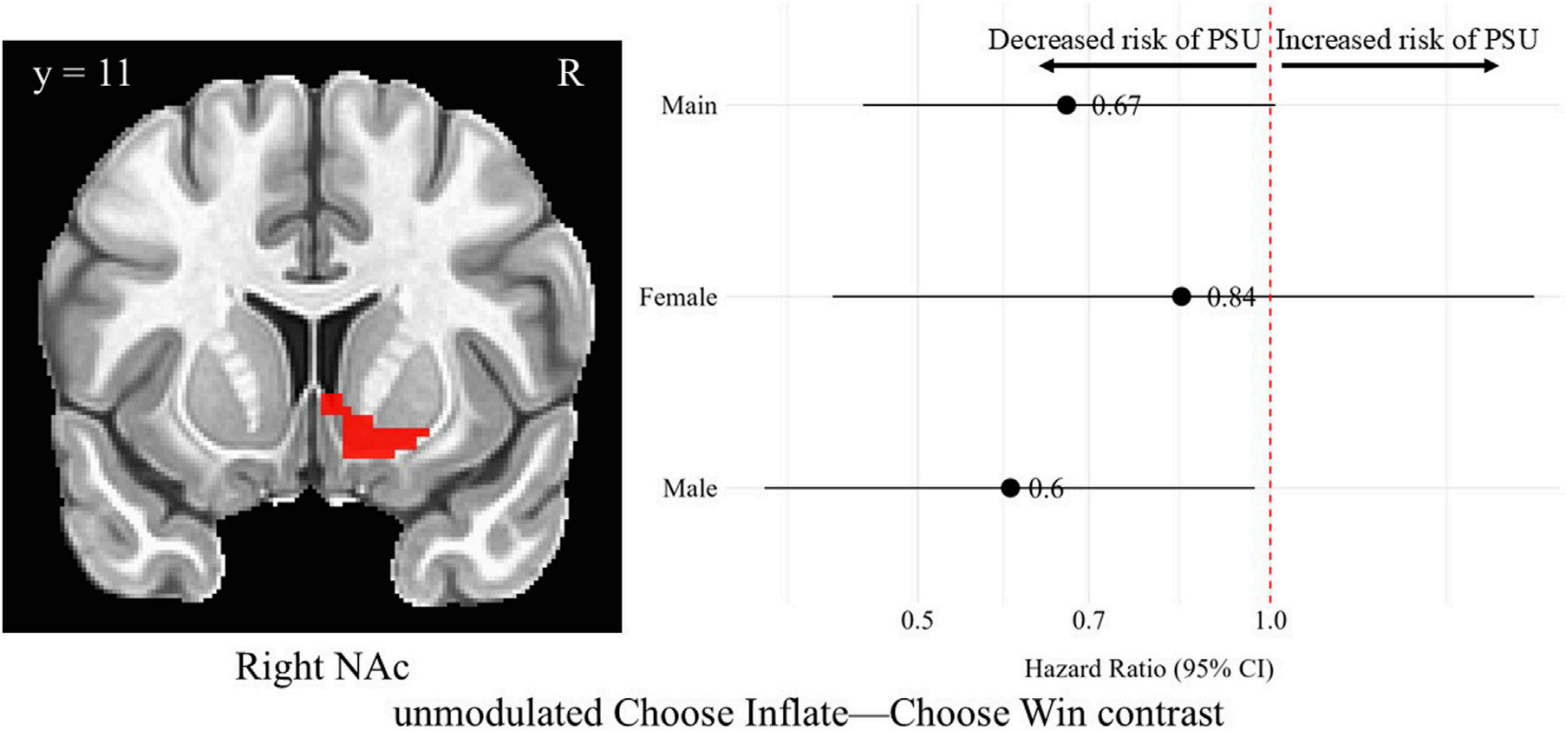
Hazard ratios for problematic substance use (PSU) risk based on unmodulated choice phase activation in the right nucleus accumbens. A coronal image of the right NAc cluster is depicted in the left side of the figure. On the right, each point represents the estimated HR for PSU risk per unit increase in activation, while the horizontal lines indicate the corresponding 95% CIs. A red vertical reference line at HR = 1.0 indicates no effect. Points to the right of this line suggest greater risk, whereas points to the left indicate lower risk of PSU. Statistically significant associations are indicated by confidence intervals that do not cross the reference line. Models were adjusted for family history of substance use, parental monitoring, and traumatic violence exposure.

In the right NAc during the Choose Inflate—Choose Win contrast, males’ unmodulated, standardized brain activation was associated with a lower hazard of problematic substance use [HR = 0.60, 95% CI (0.37, 0.97), *p* = 0.03], although this finding did not survive correction for multiple comparisons. Unmodulated brain activation in the right NAc region during the choice contrast was not significantly associated with problematic substance use for the overall sample or for females, and there was no difference between the hazard ratios for males and females for this contrast in this region. Figure 4 shows the right NAc region and a forest plot of the hazard ratios with 95% confidence intervals for the overall, female, and male samples corresponding to the relationship between average activation intensities during the unmodulated Choose Inflate—Choose Win contrast and problematic substance use.

**FIGURE 5:**
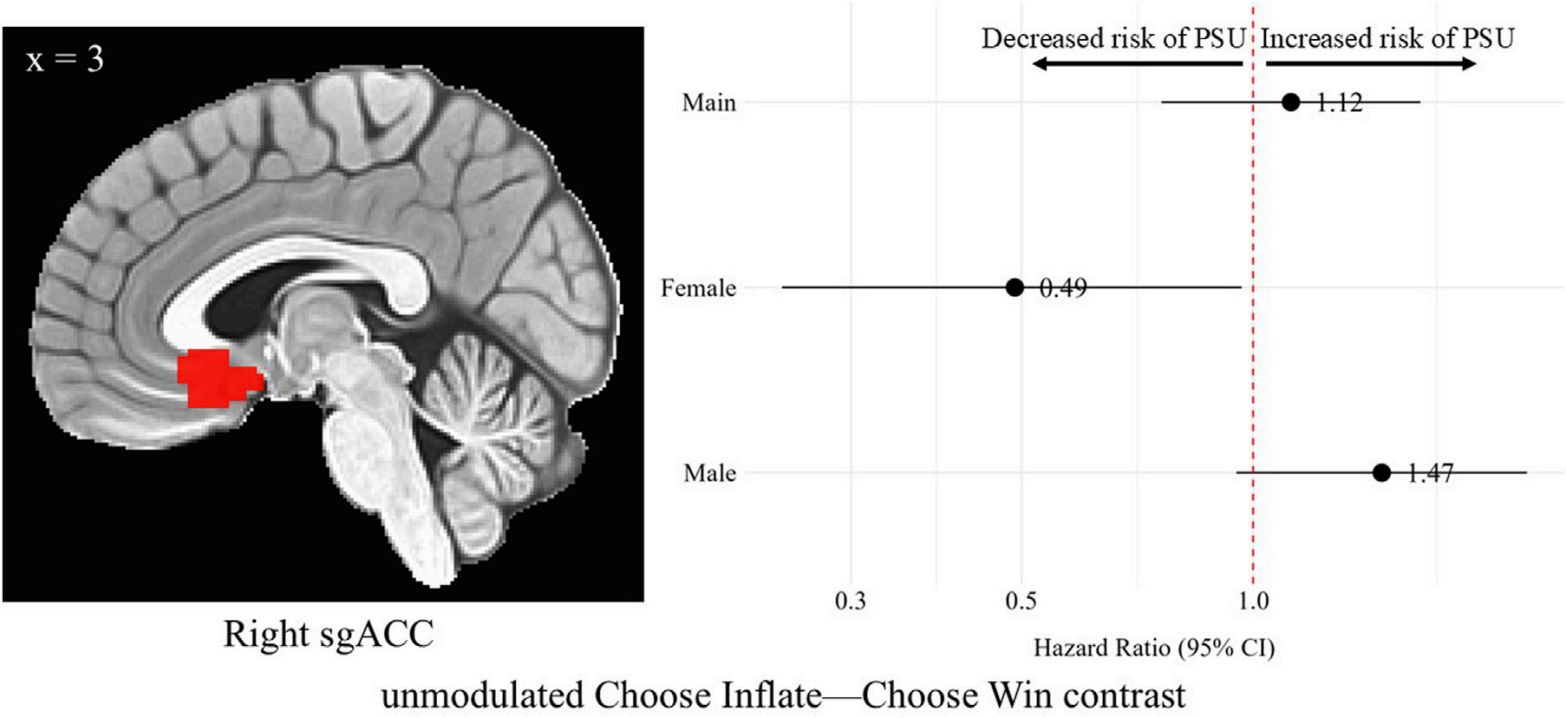
Hazard ratios for problematic substance use (PSU) risk based on unmodulated choice phase activation in the right subgenual anterior cingulate cortex. A sagittal image of the right sgACC cluster is depicted in the left side of the figure. On the right, each point represents the estimated HR for PSU risk per unit increase in activation, while the horizontal lines indicate the corresponding 95% CIs. A red vertical reference line at HR = 1.0 indicates no effect. Points to the right of this line suggest greater risk, whereas points to the left indicate lower risk of PSU. Statistically significant associations are indicated by confidence intervals that do not cross the reference line. Models were adjusted for family history of substance use, parental monitoring, and traumatic violence exposure.

In the right sgACC during the Choose Inflate—Choose Win contrast, females’ unmodulated, standardized brain activation was associated with a lower hazard of problematic substance use [HR = 0.49, 95% CI (0.24, 0.97), *p* = 0.03]. No differences in hazard of problematic substance use were observed for the overall sample or for males in this region on the unmodulated choice contrast. A difference was found when comparing the hazard ratios for males and females in the right sgACC (*p* = 0.02). However, none of these differences survived correction for multiple comparisons. Figure 5 shows the right sgACC ROI alongside a forest plot displaying the hazard ratios with 95% confidence intervals for the overall, female, and male samples corresponding to the relationship between average activation intensities during the unmodulated choice phase contrast and problematic substance use.

There were no findings with unmodulated BOLD signal in the left NAc or left sgACC.

### Outcome phase results

There were no significant uncorrected or corrected findings for the outcome phase contrasts, modulated or unmodulated.

### Secondary analysis with behavior variables results

As in the main analysis, none of the relationships between brain activation and problematic substance use, when adjusted for reward sensitivity or loss aversion, survived correction for multiple comparisons and therefore only the unadjusted results are presented and discussed below and in the supplement.

#### Behavior variables and problematic substance use

All hazard ratios and their corresponding confidence intervals for the individual behavior variables as predictors for problematic substance use in the overall sample, and in female and male subsamples, are presented in Supplementary Table S4. There were significant hazard relationships between reward sensitivity [HR = 1.76, 95% CI (1.14, 2.72), *p* = 0.01] and loss aversion [HR = 1.04, 95% CI (1.01, 1.08), *p* = 0.01] and problematic substance use in the overall sample. After breaking down by sex, these relationships seem to be driven by males as only they showed a significant relationship for both variables where greater reward sensitivity (greater average adjusted pumps) was associated with a greater hazard of problematic substance use [HR = 2.29, 95% CI (1.33, 3.93), *p* < 0.01] as was less loss aversion (greater total inflations after explosions) [HR = 1.06, 95% CI (1.02, 1.11), *p* < 0.01].

#### Reward sensitivity’s interaction with brain activation on problematic substance use hazard

For average adjusted pumps (a measure of reward sensitivity), the hazard ratios for problematic substance use and average brain activation, adjusted for reward sensitivity, are reported in Supplementary Table S5. Additionally, the hazard ratios for the interaction between brain activation and reward sensitivity are reported in Supplementary Table S5. All hazard ratios are reported for both male and female subsamples and are uncorrected for multiple comparisons. Regarding how reward sensitivity affected the relationship between brain activation during the BART and problematic substance use in females and males, there were no significant relationships for males nor females between brain activation and PSU when adjusting for reward sensitivity. However, in the right sgACC, on the modulated Outcome Explode—Outcome Inflate contrast, the interaction between activation and reward sensitivity showed a significant hazard ratio of 0.53. This indicates that the hazard associated with outcome phase activation in the right sgACC and problematic substance use decreased by 47% with every unit increase in risk sensitivity (with average adjusted pumps increasing by one). No other interaction terms in the other three ROIs nor contrasts were significant.

#### Loss aversion’s interaction with brain activation on problematic substance use hazard

The hazard ratios for brain activation when adjusting for total inflations after explosions (a measure of loss aversion), including its interaction with brain activation in predicting the hazard of problematic substance use in females and males, are presented in Supplementary Table S6. Regarding how loss aversion affected the relationship between BART brain activation and problematic substance use in females, there was a significant relationship in the left NAc during the modulated Outcome Explode—Outcome Inflate contrast where greater modulated outcome phase activation was associated with greater hazard of problematic substance use [HR = 7.68, 95% CI (1.18, 49.90), *p* = 0.03]. For females, no other ROI nor contrast was significant and no interaction term between loss aversion and brain activation was significantly related to the hazard of problematic substance use either.

For males, when loss aversion was included in the model, in the modulated Choose Inflate—Choose Win contrast, in the right NAc, greater modulated choice phase contrast was significantly associated with a smaller hazard of problematic substance use [HR = 0.11, 95% CI (0.02, 0.64), p = 0.01]. The interaction between loss aversion and modulated right NAc choice phase activation was also significant, but in the opposite direction where for every unit increase in inflations after explosions (every unit decrease in loss aversion), the hazard increased by 7% [HR = 1.07, 95% CI (1.01, 1.14), p = 0.01]. No other main nor interaction term relationships were significant in the modulated choice phase contrast for males. When loss aversion is included, greater unmodulated choice phase activation in the left sgACC was associated with a greater hazard of problematic substance use in males [HR = 6.18, 95% CI (1.22, 31.37), p = 0.02], and the interaction between loss aversion and modulated right NAc choice phase activation was also significant, but in the opposite direction where with every unit decrease in loss aversion, the hazard decreased by 5% [HR = 0.95, 95% CI (0.90, 1.00), p = 0.04]. No other main nor interaction terms were significantly related to problematic substance use for unmodulated choice phase activation in males.

When loss aversion is included in the model, greater modulated outcome phase activation in the right NAc was associated with smaller hazard of problematic substance use in males [HR = 0.26, 95% CI (0.10, 0.69), *p* < 0.01], and the interaction between loss aversion and modulated right NAc outcome phase activation was also significant, but in the opposite direction where each unit decrease in loss aversion was associated with a 7% increase in the hazard of problematic substance use [HR = 1.07, 95% CI (1.01, 1.13), p = 0.01]. There were no other signification relationships for males in modulate outcome phase contrast. Lastly, when loss aversion is included, greater unmodulated outcome phase activation in the left sgACC was associated with a greater hazard of problematic substance use in males [HR = 5.19, 95% CI (1.11, 24.31), *p* = 0.03], while the interaction between greater unmodulated outcome phase activation in the left sgACC showed that with each unit decrease in loss aversion, the hazard decreased by 5% in males [HR = 0.95, 95% CI (0.91, 1.00), *p* = 0.04]. These were the only significant relationships, main and interaction, for unmodulated outcome phase contrast activation in males.

### Stimulant medication sensitivity results

Across the 16 comparisons (4 contrasts × 4 regions of interest), only one significant difference emerged. Specifically, in the right sgACC, for the modulated Outcome Explode vs. Outcome Inflate contrast, participants not on stimulant medication exhibited lower standardized activation (M = −0.17) compared to those on medication (M = 0.22), p = 0.02. The other group averages and t-test comparisons are described in Supplementary Table S2.

No significant differences were observed between stimulant groups in terms of problematic substance use (χ^2^ = 1.44, df = 1, p = 0.23).

### Gender diverse youth supplementary analysis results

All HRs and their corresponding confidence intervals for each ROI and contrast adjusting for gender are presented in Supplementary Table S3. With the adjustment of gender, most models retained direction and significance apart from the findings in the sgACC for females with EXT disorders where the association between greater unmodulated choice phase activation and problematic substance use (HR = 0.51, 95% CI [0.26, 1.00], *p* = 0.05) became non-significant. And for modulated choice phase activation in the right NAc, the comparison between females and males’ HRs became non-significant (*p* = 0.05).

## Discussion

The goal of the current study was to investigate sex differences in associations between brain activation during risky decision-making and the emergence of problematic substance use during early adolescence in youth with EXT disorders. Two brain regions were selected for analysis due to prior evidence linking activation in these regions to decision-making in adolescence. The included regions which have been shown to be activated during decision making, and abnormally so in youth with ADHD and other SUD risk factors, included bilateral nucleus accumbens (NAc) and subgenual anterior cingulate cortex (sgACC). Our findings were all isolated to the choice phase of the decision-making process (i.e., Choose Inflate—Choose Win contrast), both parametrically modulated and unmodulated analyses. We observed that among a sample of youth at particularly high risk for the development of substance use and SUDs, by virtue of their EXT disorder diagnoses, there were sex differences in the association between both NAc and sgACC modulated and unmodulated activation during risky decision-making and problematic adolescent substance use. For females, greater activation in either of these regions was associated with a lower hazard of developing problematic substance use. For males, unmodulated activation in the NAc was associated with a lower hazard of developing problematic substance use.

Our preliminary findings emerged exclusively during the choice phase of the BART, which engages brain regions involved in deliberating and selecting between risky and safe options. In contrast, we found no significant associations during the outcome phase, which encompasses responses to the consequences of choices made and potential learning from them. Our hypotheses for the NAc were also isolated to the choice phase. While one early study in our sample identified differences in the sgACC during the outcome phase of the BART task [56], all subsequent studies examining group differences between youth with and without externalizing pathology have reported significant findings exclusively during the choice phase [17, 78, 83]. Notably, the prior investigations of sex differences also found effects limited to the choice phase [17, 83]. In the Hulvershorn 2015 study, significant group differences were observed during the outcome phase, but these were seen for comparisons between youth with EXT pathology and family history of substance use disorder, and the other groups (controls and youth with EXT pathology only) and for outcome inflate and outcome explode individually. In our current study, we controlled for family history of SUDs with a larger sample size and examined the *contrast* between outcome explode and inflate. These methodological differences may also explain why our prior publications [17, 78] observed sex or group differences, respectively, specifically during the choice phase. Moreover, to our knowledge, no previous study has examined whether brain activation during these task phases is associated with future substance use in substance-naïve adolescents. Our hypotheses were informed by evidence from at-risk groups and individuals with established substance use disorders, which may not fully translate to younger, substance-naïve populations. It remains unclear why neural activity during the choice phase—but not the outcome phase—was associated with later problematic substance use in our sample. One possibility is that deficits in deliberative or emotionally charged decision-making are more critical to future substance use vulnerability than how one reacts to feedback—particularly in a task where the feedback is financial rather than directly drug-related [113, 114]. These findings raise important questions: could the neural processing of risky decisions, rather than reactions to outcomes, be a stronger predictor of future substance use? Larger samples are needed to explore whether this pattern is consistent and potentially central to understanding the BART’s predictive utility.

Regarding the findings in the NAc, one possible explanation for these sex differences may lie in the well-studied sex differences in NAc anatomy are observed during adolescence with males exhibiting first an increase in ventral striatal volume before volume begins to decline, whereas females exhibit a steady decline throughout adolescence [115, 116]. Some sex-specific differences in how the NAc relates to SUD, particularly in relation to impulsivity, have been identified: one study found that in adolescent, alcohol-naïve males, larger NAc volume was associated with increased sensation seeking, which mediated their higher likelihood of alcohol use, while in females, NAc volume directly predicted alcohol use without mediation by sensation seeking [35]. Therefore, levels of trait impulsivity likely play a larger role in the relationship between reward network processing during decision-making and real-world substance use outcomes in males with disorders of impulsivity. Interestingly, a sex difference existed in our NAc findings across brain activation during the choice phase where only modulated brain activation was associated with lower problematic substance use in females, but for males, it was unmodulated brain activation that was associated with lower problematic substance use. In other words, greater activation in the NAc with increasing chance of balloon explosion when making a risky versus safe choice was protective against problematic substance use in females with EXT disorders, but not in males with these disorders. And on average, greater activation when making a risky over safe choice, ignoring risk of explosion, was protective against problematic substance use in males with EXT disorders, but not in females.

The NAc is typically engaged during risky choices as it plays a key role in shaping reward-driven behaviors through connecting reward experiences to emotional and motivational aspects [24, 117]. Therefore, our findings suggest that a possible explanation for sex differences in substance use outcomes may involve sex-specific maturation of the NAc, particularly in relation to reward processing. Females may derive greater benefit from appropriately ascribing emotional/motivational valence to risky decisions *as those decisions become increasingly risky* which may be a marker of a biologically protective system. Essentially, the modulated sex difference finding indicates females with EXT disorders may be appropriately calculating increasing risk and chance of poor outcome via successful attribution of emotional or affective value to risky choices. For males with EXT disorders, it appears that engaging the emotional/motivational valence-ascribing NAc during choice, regardless of the level of that choice’s riskiness, is protective against problematic substance use. Based on the female-specific findings in the NAc, a potential sex-specific clinical application could involve behavioral therapy interventions, such as that successfully implemented by our group with the Impulsive Decision Reduction Training for Youth (IDRT-Y) program [118], that target decision-making deficits. Specific to our findings, these interventions could focus on appropriately assigning emotional and motivational valence, as risk increases, during decision-making to reduce the risk of future problematic substance use, particularly for females who do not show this skill. Further, given the male-specific findings of a protective effect of NAc activation with risky versus safe choice independent of balloon size, a potential targetable therapy for males with EXT disorders could involve improving overall risk calculation and enhancing the ability to recognize signals of varying levels of risk associated with different choices during decision-making.

Our sgACC finding in the choice phase contrast showed that when females were making a risky choice over a safe choice (Choose Inflate—Choose Win), greater unmodulated activation in the right sgACC was associated with a lower hazard of developing problematic substance use (HR < 0). Had we not analyzed this relationship by sex, the association between sgACC unmodulated brain activation and problematic substance use in the overall sample would have been completely obscured (Figure 5, “Main”). Additionally, the nearly significant opposite trend in males with EXT disorders (HR > 0) would not have been identified. Other studies have found sex differences in this region during risky choice in a sample which included adolescents with conduct disorder symptoms and an SUD as well as healthy adolescents: irrespective of conduct disorder symptom/SUD or healthy control group status, all females showed greater activation than all males during risky choice in the right subgenual ACC [16]—indicating our findings *may* exist outside the context of EXT psychopathology and instead reflect a general sex difference. Atypical functional connectivity seen in ADHD may also be at play here in our sample with both ADHD and a disruptive behavior disorder: stronger positive connectivity between the striatum and both the vmPFC and the ACC has been found in children with ADHD compared to typically developing controls and was more pronounced in girls with ADHD [119]. Of note, in comparison to our sample, the group with ADHD in that study was allowed to have comorbid ODD, but comorbid CD was an exclusion criterion. These prior findings on sex differences in the sgACC associated with EXT psychopathology and reward processing taken together with our finding suggest that sgACC activation during reward processing is a biomarker that differentiates males and females who are at-risk for SUD. The sgACC has a role in avoiding loss through reward-related emotion and motivation processing [40, 41] and uncertainty/lack of confidence during decision-making [45, 47–50]. Therefore, making a risky choice would appropriately correspond with greater sgACC activation due to a signal in this region that making a risky choice could result in a negative outcome [39]. Further, this finding indicates that sex differences in substance use outcomes may be related to dysfunctional loss avoidance in males with EXT disorders when making risky choices. Specifically, their lack of protective, risk-averse behavior during decision-making may be driving the effect. In contrast, when loss avoidance functions appropriately in females during decision-making, it may serve as a protective factor against problematic substance use.

While not a sex-specific finding, the whole group overall main effect finding in the right NAc is noteworthy. In the right NAc, greater average brain activation during risky choice is associated with a lower hazard of problematic substance use. This aligns well with the existing research of the NAc’s role in risky decision-making discussed above. Additionally, individuals with family history of SUD often show blunted NAc activation during decision-making [29, 30]. This supports the idea that the relationship between NAc activation and problematic substance use observed here may be more related to sensation seeking and EXT psychopathology rather than the genetic risk of family history of SUD (which was controlled for in this study) [31]. Furthermore, research on ADHD, an EXT disorder, has demonstrated hypoconnectivity between the prefrontal cortex and NAc [25–27], which may be part of the explanation for blunted NAc activation during risky decision-making in EXT psychopathology.

As a secondary analysis, we explored how behavioral markers of reward sensitivity (average adjusted pumps) and loss aversion (total inflations after explosions) related to the hazard of developing problematic substance use. In the overall sample, there were significant relationships between each variable and problematic substance use which seems to be driven by males, who, like the significant finding from the overall sample, showed greater reward sensitivity and lower loss aversion independently and understandably associated with a higher hazard of problematic substance use. These relationships were not significant in females. These results suggest potential sex-specific behavioral risk pathways.

We also examined how these behavioral traits moderated the relationship between BART-related brain activation and problematic substance use. Interestingly, when adjusting for reward sensitivity, the effects observed in the modulated and unmodulated choice phase contrast were no longer significant. And then the only significant finding with brain activation and reward sensitivity was the interaction between modulated outcome phase contrast activation where greater activation in the right sgACC with explosions vs. inflations was associated with lower hazard of problematic substance use for those with greater reward sensitivity in males only. For the loss aversion measure in males, several brain-behavior interactions emerged which were unique compared to the analysis unadjusted for loss aversion. For males, greater modulated choice phase and outcome phase activation among those with less aversion to loss, was associated with a greater hazard of problematic substance use, while for unmodulated activation not accounting for increased risk of balloon explosion, the opposite effect was true of lower hazard of problematic substance use. Our finding that the behavioral measure of loss aversion moderates the relationship between brain activation and problematic substance use only in males may be related to well-documented sex differences in loss aversion, with females typically exhibiting greater risk aversion [72–74]; one proposed explanation is that these sex differences in loss aversion and optimism may stem from a combination of evolutionary pressures—such as inter-male competition—and cultural influences, including societal norms that shape risk-related behaviors [72]. These findings highlight the nuanced interplay between individual behavioral traits and neural processing in shaping substance use vulnerability, particularly in males.

When evaluating differences in decision-making neural activation between those using stimulant medication or not, we found that in the right sgACC, for the modulated outcome phase contrast, participants not on stimulant medication exhibited lower standardized activation. With the sgACC’s role in affective valuation, emotional regulation, and integrating reward signals over time [42–46], the differential activation in this region between adolescents using or not using stimulant medication may reflect a couple aspects of stimulant use in adolescents with EXT psychopathology. First, it is possible, though not testable within the scope of this study, that individuals on stimulant medication at baseline were prescribed these medications due to previously elevated emotional lability; in other words, part of their externalizing profile may have included pre-existing heightened emotional reactivity. The modulatory effects of stimulant medication on emotional reactivity. Second, amphetamine derivatives have been associated with increased risk or irritability and emotional lability [120, 121], which may explain why those on stimulant medication at baseline (imaging day) showed greater sgACC reactivity to the negative cue of explosion vs. inflation, given the region’s role in processing negative emotion. The effect emerging specifically during the outcome phase—rather than the choice phase—could reflect links to emotional lability. Notably, no group differences in problematic substance use suggest that neural differences were not driven by substance-related factors. Still, given the exploratory nature and limited significant findings, the potential influence of stimulant use on neural risk processing and substance use outcomes warrants further study.

Adjusting for gender marginally influenced the results, which emphasizes the need for further exploration of how gender diversity interacts with the relationships analyzed here. The observed changes may be partly due to an imbalanced sample—possibly resulting in statistical artifacts, which may be further evidenced by the small magnitude of statistical changes seen in the HRs and *p*-values in the adjusted models. Prior research has established that gender diverse individuals are more vulnerable to EXT behaviors and ADHD [98, 100, 101] as well as to SUDs and other risky behaviors [97–99]. However, there is a gap in the research examining how deficits in risky decision-making neural processing may underlie the increased impulsive and risky behaviors observed among gender diverse people.

This study has some limitations. One limitation of this study is that problematic substance use was derived from self and guardian-report data, as urine drug screens were not collected from all participants during the COVID-19 pandemic. This self-reporting of substance use carries potential biases like social desirability, memory and recall issues, underreporting for protection, and lack of objective verification [122–124], which could compromise the accuracy and reliability of the data. However, the inclusion of both guardian and child reports may mitigate some of these biases and offer a more comprehensive and accurate representation. Further, adolescent self-report tends to align closely with objective measures like urinalysis [125]. Parent reports show fair-to-good agreement with youth reports, particularly for use/non-use and frequency of common substances such as alcohol and cannabis—substances and level of reporting most relevant to our sample [125, 126]. Youth self-report has been shown to be reliable and valid for the commonly used substances used in our sample [125, 127], while parents often underreport [125]. Therefore, if a parent in this study reported their adolescent’s problematic substance use, it likely reflects genuine adolescent use. Another limitation relates to the fact that some members of our sample, with diagnosed EXT disorders, have previously and throughout the study taken psychostimulant medications, which could potentially affect brain activation and substance use behaviors. Across our participants who have taken medication, dosage and the oral method of administration differs greatly from those typically associated with stimulant abuse. With that, fewer than one-third of ADHD participants were taking medication at baseline, and stimulant medication was withheld for at least 24 h before the imaging session and subjects were excluded who did not hold medication before baseline visits (see Figure 1). Although medication effects could influence these results, participants were not under the immediate effects of psychostimulant medication during their baseline behavioral assessments and brain imaging sessions and recruiting a sample with EXT disorders entirely naive to psychotropic drugs would have been quite unrealistic and unrepresentative. Another limitation of this study related to the smaller sample size is the unbalanced sex distribution, with fewer females than males. While this ratio reflects the overall population of individuals with EXT disorders [128–130], which guided our sampling approach, the uneven gender distribution may limit our ability to fully examine sex differences. To better explore sex differences in future research, a larger sample and oversampling females with EXT disorders would be beneficial. Although, a related strength of this sample, and a key aspect of its novelty, is that we are able to offer an investigation into sex differences in substance use risk within a population where previous studies were not able to examine the effects of sex: subjects with clinician-diagnosed ADHD and a disruptive behavior disorder.

In summary, we believe this study provides novel insights into sex differences in the relationships between neural activation during risky decision-making and problematic substance use among particularly high-risk youth. To summarize, greater activation in the right NAc when making a risky versus safe choice, as that choice became riskier, was associated with a lower hazard of problematic substance use in the overall sample and in females. Our NAc findings suggest that intact motivational valence ascription during risky decision-making is a function which may be protective in youth with EXT disorders against problematic substance use. Similarly, we found that greater unmodulated activation in the right sgACC during the choice phase was associated with a lower hazard of problematic substance use in females only. Our sgACC findings suggest that functions which are protective in females with EXT disorders against problematic substance use (i.e., proper loss avoidance), may either not hold the same protective value for males with EXT disorders or may be dysfunctional in males. Future directions for this work, in addition to replication with a larger sample size, include evaluating whether measures of sensation seeking and impulsivity influence the relationship between brain activation during risky decision-making and substance use outcomes, as well as exploring the role of sex in this relationship. In conclusion, these findings highlight specific cognitive, emotional, and behavioral targets when developing sex-specific interventions for SUD during adolescence.

## Summary

Adolescents with externalizing (EXT) disorders, such as ADHD and conduct disorder, are at heightened risk for substance use disorders (SUDs), partly due to impairments in risky decision-making. However, most studies have focused on male participants or failed to consider sex differences. Neuroimaging research suggests that females with EXT disorders may exhibit distinct brain activation patterns in regions linked to risky decision-making. This study explores how sex differences risky decision-making brain activation are associated with SUD risk in youth with EXT disorders.

Data were collected from 115 drug-naive youth with EXT psychopathology (78 males and 37 females) as part of an ongoing longitudinal study. Participants completed an fMRI-compatible Balloon Analogue Risk Task (BART) to assess brain activation during risky decision-making, accounting for the probability of balloon explosion. They were followed every 6 months for up to 84 months to track problematic substance use. Statistical analysis examined sex differences in hazard of problematic substance use based on risky decision-making brain activation.

Results revealed that higher modulated activation in the right nucleus accumbens (NAc) during the Choose Inflate—Choose Win contrast (a risky over safe decision as that decision became riskier) was associated with a lower hazard of problematic substance use in the overall sample [Hazard Ratio (HR) = 0.68, 95% CI (0.49, 0.94)] and in females specifically, but not in males. Similarly, higher unmodulated activation in the right subgenual anterior cingulate cortex (sgACC) during the choice contrast was associated with a lower hazard of problematic substance use in females [HR = 0.49, 95% CI (0.24, 0.97)]. There was a significant difference between the hazard ratios for males and females in both regions. In the right nucleus accumbens, higher unmodulated choice contrast activation in males was associated with lower hazard of problematic substance use [HR = 0.60, 95% CI (0.37, 0.97)].

These findings suggest that greater activation in the NAc and sgACC during risky decision-making may protect against problematic substance use, particularly in females. The study highlights sex differences in neural mechanisms of reward processing and loss avoidance, indicating the need for sex-specific interventions targeting these processes in youth at risk for SUDs.

## Data Availability

The datasets presented in this article are not readily available because the dataset will be shared via NIH data sharing platforms. Requests to access the datasets should be directed to LH, lhulvers@iu.edu.
