# Supplementary material for: Sex differences in reward network activation are linked to problematic substance use among high-risk adolescents
Source: Adv Drug Alcohol Res. 2025 Dec 4;5:14591. doi: 10.3389/adar.2025.14591 (PMC12711589; doi:10.3389/adar.2025.14591)
Supplement: Supplementary file 1 [file DataSheet1.docx]

**Supplement 1**

***Potential Covariates not included in final model:***

Socioeconomic Status (SES)

SES was approximated by parental education as a possible covariate as low parental education has been identified as a risk factor for drug abuse among adolescents (1). At baseline, guardians were asked the highest degree/level of school the child’s mother and father completed. For this analysis, the SES variable was coded as the highest/max education obtained by either biological parent in a factor variable of 1) less than high school, 2) high school, 3) some college or college, 4) some graduate or graduate school, or 5) unknown. Data missingness for SES was handled with multiple imputations utilizing the MICE (Multivariate Imputation by Chained Equations) package in R (2) which employed a proportional odds logistic regression (POLR) model to impute SES accounting for family history of substance use disorder (SUD) and sex.

***Specific reasons for n=44 unsatisfactory functional imaging participant exclusions:***

First, eight subjects were unable to complete the entire three runs of BART due to visibly excessive motion/restlessness, talking during the task, taking breaks in the imaging session for things like using the restroom, or in one case, a fire drill. These subjects’ data were not analyzed and thus they were excluded (n=8). In the first phase of imaging quality evaluation of those who completed three BART runs, participants showing motion artifacts during the anatomical scan, which led to problems with volume registration, were excluded from the analysis (n = 11). Then, further visual inspection of brain activity was conducted to ensure the presence of expected activation patterns corresponding to the task. This involved inspecting the spatial distribution of activation maps to ensure engagement of typical task-related regions, such as the visual cortex. Participants were excluded if expected activation in regions like the visual cortex was absent (n = 5), or if there was widespread negative activation, indicating possible artifacts from global signal regression (n = 7). Lastly, if the activation distribution in the maps appeared excessively diffuse, non-localized, or lacked a clear pattern, this was attributed to motion artifacts, and participants with confirmed high motion were excluded (n = 13). As a result, a total of 44 participants were excluded due to suboptimal functional imaging.

***Covariate Selection Results***

Akaike Information Criterion (AIC) stepwise model selection revealed the optimal, simplified model predicting problematic substance use included the z-score of average brain activation, sex, family history of SUD, the traumatic violence subscale of the Screen for Violence Exposure (SAVE), and the average score of parental monitoring. Each covariate besides family history of SUD was present in every model (as shown in Table S1 below), and due to family history of SUD’s well-established genetic liability for SUD (3, 4), we included it as a covariate in the final model.

Table S1. Unadjusted and Adjusted AIC for Each Contrast and Region of Interest (ROI)

| Contrast | ROI | Unadj. AIC | Adj. AIC | Sex | FH of SUD | SES | SAVE TV | SAVE IV | PM |
| --- | --- | --- | --- | --- | --- | --- | --- | --- | --- |
| CICW_mod | left sgACC | 248.04 | 238.59 | 1 | 0 | 0 | 1 | 0 | 1 |
| CICW_mod | left NAc | 249.47 | 238.67 | 1 | 0 | 0 | 1 | 0 | 1 |
| CICW_mod | right sgACC | 248.93 | 238.32 | 1 | 0 | 0 | 1 | 0 | 1 |
| CICW_mod | right NAc | 243.71 | 232.92 | 1 | 0 | 0 | 1 | 0 | 1 |
| CICW_unmod | left sgACC | 248.18 | 238.05 | 1 | 0 | 0 | 1 | 0 | 1 |
| CICW_unmod | left NAc | 249.64 | 238.80 | 1 | 1 | 0 | 1 | 0 | 1 |
| CICW_unmod | right sgACC | 249.39 | 238.48 | 1 | 0 | 0 | 1 | 0 | 1 |
| CICW_unmod | right NAc | 247.24 | 234.74 | 1 | 0 | 0 | 1 | 0 | 1 |
| OEOI_mod | left sgACC | 249.00 | 238.17 | 1 | 0 | 0 | 1 | 0 | 1 |
| OEOI_mod | left NAc | 248.60 | 237.79 | 1 | 1 | 0 | 1 | 0 | 1 |
| OEOI_mod | right sgACC | 249.59 | 238.36 | 1 | 1 | 0 | 1 | 0 | 1 |
| OEOI_mod | right NAc | 248.38 | 237.45 | 1 | 1 | 0 | 1 | 0 | 1 |
| OEOI_unmod | left sgACC | 247.39 | 238.63 | 1 | 0 | 0 | 1 | 0 | 1 |
| OEOI_unmod | left NAc | 249.70 | 238.80 | 1 | 1 | 0 | 1 | 0 | 1 |
| OEOI_unmod | right sgACC | 249.37 | 238.81 | 1 | 1 | 0 | 1 | 0 | 1 |
| OEOI_unmod | right NAc | 249.24 | 237.87 | 1 | 0 | 0 | 1 | 0 | 1 |

FH of SUD = family history of substance use disorder, SES = socioeconomic status, proxied here with maximum parental education, SAVE TV = traumatic violence exposure, SAVE IV = interpersonal violence exposure, PM= parental monitoring; NAc = nucleus accumbens, sgACC = subgenual anterior cingulate cortex, CICW = Choose Inflate—Choose Win contrast, OEOI = Outcome—Outcome Inflate contrast, mod = parametrically modulated activation with probability of balloon explosion, unmod = not parametrically modulated/averaged across all events; 1 = that covariate was included in the adjusted model for that ROI/contrast, 0 = that covariate was *not* included in the adjusted model for that ROI/contrast

Table S2. Sensitivity analysis of average standardized activation by stimulant medication status at baseline

| ROI | Contrast | No Stimulants Mean | Stimulants Mean | *p*-value |
| --- | --- | --- | --- | --- |
| Left NAc | CICW_mod | 0.06 | -0.07 | 0.48 |
| Left sgACC | CICW_mod | -0.03 | 0.03 | 0.73 |
| Right NAc | CICW_mod | -0.11 | 0.14 | 0.16 |
| Right sgACC | CICW_mod | -0.01 | 0.01 | 0.92 |
| Left NAc | CICW_unmod | -0.05 | 0.06 | 0.58 |
| Left sgACC | CICW_unmod | -0.01 | 0.02 | 0.86 |
| Right NAc | CICW_unmod | -0.05 | 0.07 | 0.51 |
| Right sgACC | CICW_unmod | 0.06 | -0.07 | 0.49 |
| Left NAc | OEOI_mod | -0.09 | 0.11 | 0.24 |
| Left sgACC | OEOI_mod | -0.10 | 0.13 | 0.22 |
| Right NAc | OEOI_mod | -0.03 | 0.04 | 0.71 |
| Right sgACC | OEOI_mod | -0.17 | 0.22 | 0.02 |
| Left NAc | OEOI_unmod | -0.11 | 0.14 | 0.19 |
| Left sgACC | OEOI_unmod | 0.11 | -0.14 | 0.15 |
| Right NAc | OEOI_unmod | -0.04 | 0.05 | 0.66 |
| Right sgACC | OEOI_unmod | 0.09 | -0.11 | 0.28 |

Table S3. Supplementary analysis hazard ratios (HR) for activation (standardized) and problematic substance use adjusted for gender

| ROI | Main HR (95% CI) | *p* | Female HR  (95% CI) | *p* | Male HR  (95% CI) | *p* | Sex Diff. HR *p* |
| --- | --- | --- | --- | --- | --- | --- | --- |
| Choose Inflate—Choose Win modulated | | | | | | |  |
| Left NAc | 0.96 (0.63-1.46) | 0.83 | 0.83 (0.47-1.46) | 0.51 | 1.12 (0.63-2.01) | 0.69 | 0.75 |
| Right NAc | 0.66 (0.47-0.94) | 0.02 | 0.62 (0.41-0.94) | 0.02 | 0.75 (0.42-1.35) | 0.34 | 0.05 |
| Left sgACC | 0.93 (0.62-1.39) | 0.71 | 1.12 (0.59-2.11) | 0.73 | 0.83 (0.5-1.37) | 0.45 | 0.71 |
| Right sgACC | 0.93 (0.66-1.3) | 0.65 | 0.93 (0.6-1.44) | 0.73 | 0.92 (0.56-1.53) | 0.75 | 0.9 |
| Choose Inflate—Choose Win unmodulated | | | | | | | |
| Left NAc | 1.02 (0.68-1.52) | 0.92 | 1.13 (0.48-2.69) | 0.78 | 0.99 (0.61-1.59) | 0.95 | 0.96 |
| Right NAc | 0.68 (0.45-1.03) | 0.06 | 0.94 (0.48-1.86) | 0.85 | 0.57 (0.34-0.93) | 0.02 | 0.08 |
| Left sgACC | 1.12 (0.75-1.67) | 0.58 | 0.72 (0.38-1.36) | 0.31 | 1.43 (0.88-2.31) | 0.14 | 0.21 |
| Right sgACC | 1.13 (0.77-1.65) | 0.53 | 0.51 (0.26-1) | 0.05 | 1.48 (0.97-2.27) | 0.07 | 0.02 |
| Outcome Explode—Outcome Inflate modulated | | | | | | | |
| Left NAc | 1.26 (0.73-2.17) | 0.39 | 2.26 (0.89-5.75) | 0.08 | 0.95 (0.58-1.58) | 0.85 | 0.23 |
| Right NAc | 1.24 (0.81-1.9) | 0.32 | 1.64 (0.92-2.92) | 0.09 | 0.83 (0.44-1.57) | 0.56 | 0.21 |
| Left sgACC | 0.92 (0.63-1.33) | 0.65 | 1.15 (0.66-2) | 0.62 | 0.77 (0.46-1.28) | 0.31 | 0.54 |
| Right sgACC | 0.88 (0.64-1.23) | 0.46 | 1.21 (0.44-3.32) | 0.71 | 0.85 (0.63-1.16) | 0.32 | 0.57 |
| Outcome Explode—Outcome Inflate unmodulated | | | | | | | |
| Left NAc | 1.02 (0.67-1.56) | 0.92 | 0.97 (0.49-1.94) | 0.93 | 1.05 (0.62-1.78) | 0.85 | 0.98 |
| Right NAc | 1.11 (0.78-1.58) | 0.56 | 1.11 (0.65-1.9) | 0.71 | 1.11 (0.69-1.79) | 0.66 | 0.84 |
| Left sgACC | 1.05 (0.78-1.42) | 0.73 | 1.01 (0.72-1.41) | 0.96 | 1.34 (0.65-2.74) | 0.43 | 0.73 |
| Right sgACC | 0.99 (0.76-1.3) | 0.95 | 0.99 (0.74-1.34) | 0.97 | 0.98 (0.47-2.02) | 0.95 | 0.99 |

Models were adjusted for gender (cisgender = 0, gender diverse = 1), family history of substance use, parental monitoring, and traumatic violence exposure; NAc = nucleus accumbens, sgACC = subgenual anterior cingulate cortex; problematic substance use is defined by experiencing two or more consequences for at least one substance as reported by child or guardian; sex diff HR *p* = the p-value indicating whether the hazard ratio (HR) differs significantly between males and females

Table S4. Secondary analysis: unadjusted hazard ratios (HR) between reward sensitivity, loss aversion, and problematic substance use in the overall sample, female, and male subsamples

| Behavior Variable | HR for PSU (95% CI) | *p* | HR for PSU in Females (95% CI) | *p* | HR for PSU in Males (95% CI) | *p* |
| --- | --- | --- | --- | --- | --- | --- |
| Average Adjusted Pumps (Reward Sensitivity) | 1.76 (1.14-2.72) | 0.01 | 1.13 (0.59-2.15) | 0.72 | 2.29 (1.33-3.93) | < 0.01 |
| Total Inflations after Explosions (Loss Aversion) | 1.04 (1.01-1.08) | 0.01 | 1.02 (0.96-1.07) | 0.55 | 1.06 (1.02-1.11) | < 0.01 |

Problematic substance use is defined by experiencing two or more consequences for at least one substance as reported by child or guardian

Table S5. Secondary analysis: hazard ratios (HR) for standardized brain activation predicting problematic substance use, adjusted for and interacting with reward sensitivity in male and female subsamples

| ROI | Adjusted Brain Activation HR in Females (95% CI) | *p* | Brain Activation:Average Adjusted Pumps HR in Females  (95% CI) | *p* | Adjusted Brain Activation HR in Males (95% CI) | *p* | Brain Activation:Average Adjusted Pumps HR in Males  (95% CI) | *p* |
| --- | --- | --- | --- | --- | --- | --- | --- | --- |
| Choose Inflate—Choose Win modulated | | | | | | |  |  |
| Left NAc | 2.37 (0.15-38.36) | 0.54 | 0.77 (0.39-1.5) | 0.43 | 0.02 (0-3.51) | 0.13 | 2.15 (0.84-5.48) | 0.10 |
| Right NAc | 0.96 (0.1-9.74) | 0.97 | 0.86 (0.47-1.57) | 0.62 | 0.03 (0-1.18) | 0.06 | 1.89 (0.95-3.76) | 0.07 |
| Left sgACC | 0.74 (0.01-46.9) | 0.88 | 1.14 (0.5-2.57) | 0.75 | 0.01 (0-1.27) | 0.06 | 2.44 (0.96-6.22) | 0.06 |
| Right sgACC | 0.67 (0.11-4.27) | 0.67 | 1.06 (0.72-1.56) | 0.76 | 0.14 (0-12.12) | 0.38 | 1.53 (0.64-3.67) | 0.34 |
| Choose Inflate—Choose Win unmodulated | | | | | | | |  |
| Left NAc | 21.34 (0.01-46336.16) | 0.43 | 0.57 (0.13-2.42) | 0.44 | 1.28 (0.03-51.96) | 0.89 | 0.96 (0.5-1.82) | 0.89 |
| Right NAc | 2.31 (0.01-383.82) | 0.74 | 0.82 (0.31-2.18) | 0.69 | 0.3 (0.02-5.57) | 0.41 | 1.16 (0.71-1.89) | 0.55 |
| Left sgACC | 0.03 (0-5.6) | 0.18 | 2.12 (0.71-6.37) | 0.17 | 17.63 (0.5-616.56) | 0.11 | 0.61 (0.32-1.17) | 0.13 |
| Right sgACC | 0.19 (0-28.02) | 0.51 | 1.17 (0.4-3.45) | 0.77 | 9.01 (0.42-195.46) | 0.16 | 0.68 (0.38-1.22) | 0.19 |
| Outcome Explode—Outcome Inflate modulated | | | | | | | |  |
| Left NAc | 15.56 (0.26-938.09) | 0.18 | 0.67 (0.25-1.79) | 0.42 | 1.58 (0.03-100.23) | 0.82 | 0.87 (0.36-2.08) | 0.75 |
| Right NAc | 2.31 (0.17-30.98) | 0.52 | 0.94 (0.49-1.81) | 0.84 | 0.08 (0-4.89) | 0.22 | 1.63 (0.67-3.96) | 0.28 |
| Left sgACC | 51.57 (0.9-2957.57) | 0.05 | 0.47 (0.22-1.05) | 0.06 | 5.35 (0.64-44.44) | 0.12 | 0.67 (0.43-1.06) | 0.08 |
| Right sgACC | 19.2 (0.05-7437.02) | 0.33 | 0.59 (0.16-2.2) | 0.43 | 5.45 (0.67-44.12) | 0.11 | 0.53 (0.30-0.94) | 0.03 |
| Outcome Explode—Outcome Inflate unmodulated | | | | | | | |  |
| Left NAc | 0.1 (0-3.23) | 0.19 | 1.68 (0.79-3.56) | 0.17 | 3.63 (0.23-57.89) | 0.36 | 0.79 (0.48-1.3) | 0.35 |
| Right NAc | 0.09 (0-21.94) | 0.39 | 1.77 (0.51-6.14) | 0.36 | 1.52 (0.08-28.23) | 0.77 | 0.94 (0.55-1.61) | 0.81 |
| Left sgACC | 2.26 (0.68-7.57) | 0.18 | 0.83 (0.61-1.13) | 0.22 | 15.41 (0.47-501.54) | 0.12 | 0.63 (0.34-1.19) | 0.15 |
| Right sgACC | 1.61 (0.6-4.35) | 0.34 | 0.91 (0.67-1.24) | 0.54 | 0.47 (0-51.61) | 0.75 | 1.15 (0.48-2.76) | 0.75 |

Models were adjusted for reward sensitivity, family history of substance use, parental monitoring, and traumatic violence exposure; NAc = nucleus accumbens, sgACC = subgenual anterior cingulate cortex; problematic substance use is defined by experiencing two or more consequences for at least one substance as reported by child or guardian

Table S6. Secondary analysis: hazard ratios (HR) for standardized brain activation predicting problematic substance use, adjusted for and interacting with loss aversion in male and female subsamples

| ROI | Adjusted Brain Activation HR in Females (95% CI) | *p* | Brain Activation:Total Inflations after Explosions HR in Females  (95% CI) | *p* | Adjusted Brain Activation HR in Males (95% CI) | *p* | Brain Activation:Total Inflations After Explosions HR in Males  (95% CI) | *p* |
| --- | --- | --- | --- | --- | --- | --- | --- | --- |
| Choose Inflate—Choose Win modulated | | | | | | |  |  |
| Left NAc | 0.7 (0.18-2.64) | 0.59 | 1 (0.94-1.07) | 0.92 | 1 (0.23-4.33) | 0.99 | 1.01 (0.96-1.06) | 0.66 |
| Right NAc | 0.49 (0.24-1.03) | 0.05 | 1 (0.96-1.05) | 0.99 | 0.11 (0.02-0.64) | 0.01 | 1.07 (1.01-1.14) | 0.01 |
| Left sgACC | 1.71 (0.18-16.38) | 0.64 | 0.99 (0.91-1.08) | 0.82 | 0.26 (0.04-1.53) | 0.13 | 1.04 (0.99-1.09) | 0.16 |
| Right sgACC | 0.98 (0.28-3.4) | 0.97 | 0.99 (0.94-1.04) | 0.68 | 0.81 (0.2-3.26) | 0.76 | 1.01 (0.96-1.05) | 0.71 |
| Choose Inflate—Choose Win unmodulated | | | | | | | |  |
| Left NAc | 0.46 (0.01-21.87) | 0.69 | 1.03 (0.92-1.15) | 0.63 | 1.69 (0.41-7.07) | 0.47 | 0.99 (0.95-1.03) | 0.47 |
| Right NAc | 0.41 (0.04-4.58) | 0.47 | 1.03 (0.95-1.11) | 0.52 | 0.71 (0.18-2.84) | 0.62 | 1 (0.96-1.03) | 0.86 |
| Left sgACC | 1.33 (0.26-6.72) | 0.73 | 0.99 (0.93-1.05) | 0.66 | 6.18 (1.22-31.37) | 0.02 | 0.95 (0.90-1.00) | 0.04 |
| Right sgACC | 0.34 (0.05-2.52) | 0.28 | 1.01 (0.93-1.09) | 0.88 | 2.23 (0.54-9.22) | 0.26 | 0.98 (0.94-1.03) | 0.47 |
| Outcome Explode—Outcome Inflate modulated | | | | | | | |  |
| Left NAc | 7.68 (1.18-49.90) | 0.03 | 0.96 (0.88-1.05) | 0.35 | 0.8 (0.45-1.45) | 0.46 | 1.01 (0.97-1.06) | 0.50 |
| Right NAc | 2.78 (0.96-8) | 0.05 | 0.98 (0.92-1.04) | 0.45 | 0.26 (0.10-0.69) | < 0.01 | 1.07 (1.01-1.13) | 0.01 |
| Left sgACC | 3.01 (0.31-29.22) | 0.34 | 0.97 (0.88-1.06) | 0.47 | 1.08 (0.33-3.51) | 0.9 | 0.99 (0.94-1.04) | 0.63 |
| Right sgACC | 1.57 (0.07-34.78) | 0.77 | 1.02 (0.88-1.18) | 0.77 | 0.92 (0.56-1.53) | 0.76 | 0.96 (0.91-1.02) | 0.15 |
| Outcome Explode—Outcome Inflate unmodulated | | | | | | | |  |
| Left NAc | 1.23 (0.26-5.78) | 0.79 | 0.99 (0.93-1.05) | 0.70 | 2.85 (0.89-9.18) | 0.07 | 0.96 (0.93-1) | 0.05 |
| Right NAc | 2.27 (0.66-7.85) | 0.19 | 0.96 (0.9-1.03) | 0.23 | 1.59 (0.46-5.49) | 0.46 | 0.99 (0.95-1.03) | 0.54 |
| Left sgACC | 1.77 (0.83-3.77) | 0.13 | 0.97 (0.92-1.01) | 0.17 | 5.19 (1.11-24.31) | 0.03 | 0.95 (0.91-1.00) | 0.04 |
| Right sgACC | 1.52 (0.81-2.86) | 0.19 | 0.98 (0.94-1.02) | 0.26 | 1.27 (0.28-5.85) | 0.76 | 0.99 (0.94-1.04) | 0.68 |

Models were adjusted for loss aversion, family history of substance use, parental monitoring, and traumatic violence exposure; NAc = nucleus accumbens, sgACC = subgenual anterior cingulate cortex; problematic substance use is defined by experiencing two or more consequences for at least one substance as reported by child or guardian

Bibliography

1. Nawi AM, Ismail R, Ibrahim F, Hassan MR, Manaf MRA, Amit N, et al. Risk and protective factors of drug abuse among adolescents: a systematic review. BMC Public Health (2021) 21:2088. doi: 10.1186/s12889-021-11906-2

2. Buuren Sv, Groothuis-Oudshoorn K. Multivariate Imputation by Chained Equations (2023). <https://github.com/amices/mice>

3. Merikangas KR, Stolar M, Stevens DE, Goulet J, Preisig MA, Fenton B, et al. Familial transmission of substance use disorders. Arch Gen Psychiatry (1998) 55:11. p. 973-9. doi: 10.1001/archpsyc.55.11.973

4. Steinhausen HC, Jakobsen H, Munk-Jørgensen P. Family aggregation and risk factors in substance use disorders over three generations in a nation-wide study. PLoS One (2017) 12:5. p. e0177700. doi: 10.1371/journal.pone.0177700
